# A Computational Mechanism for Unified Gain and Timing Control in the Cerebellum

**DOI:** 10.1371/journal.pone.0033319

**Published:** 2012-03-13

**Authors:** Tadashi Yamazaki, Soichi Nagao

**Affiliations:** Laboratory for Motor Learning Control, RIKEN Brain Science Institute, Wako, Saitama, Japan; Tokyo Medical and Dental University, Japan

## Abstract

Precise gain and timing control is the goal of cerebellar motor learning. Because the basic neural circuitry of the cerebellum is homogeneous throughout the cerebellar cortex, a single computational mechanism may be used for simultaneous gain and timing control. Although many computational models of the cerebellum have been proposed for either gain or timing control, few models have aimed to unify them. In this paper, we hypothesize that gain and timing control can be unified by learning of the complete waveform of the desired movement profile instructed by climbing fiber signals. To justify our hypothesis, we adopted a large-scale spiking network model of the cerebellum, which was originally developed for cerebellar timing mechanisms to explain the experimental data of Pavlovian delay eyeblink conditioning, to the gain adaptation of optokinetic response (OKR) eye movements. By conducting large-scale computer simulations, we could reproduce some features of OKR adaptation, such as the learning-related change of simple spike firing of model Purkinje cells and vestibular nuclear neurons, simulated gain increase, and frequency-dependent gain increase. These results suggest that the cerebellum may use a single computational mechanism to control gain and timing simultaneously.

## Introduction

Smooth and coordinated movements are achieved by controlling movements of different body parts precisely in both space and time. The spatial information—the amplitude or velocity of movements—is technically called “gain”, whereas the temporal information—the initiation and termination of movements—is called “timing”. Our daily movements are thus executed under precise gain and timing control. The cerebellum seems to play an essential role in the acquisition and maintenance of this gain and timing information, because patients with cerebellar diseases very often show dysmetria or delays in movement onsets. The cerebellar mechanisms for gain and timing control have typically been studied independently using two different experimental paradigms, i.e., gain adaptation of the vestibulo-ocular reflex (VOR) or optokinetic response (OKR) eye movements (e.g., [1], [2]), and timing learning in the Pavlovian delay eyeblink conditioning (e.g., [3], [4]). Correspondingly, a number of computational models of the cerebellum have been proposed independently for either VOR/OKR adaptation [5]–[12] or eyeblink conditioning [13]–[27]. Few models, however, can address both of these. Microzones and microcomplexes, which are homogeneous structures within the cerebellum, are supposed to be the basic functional unit of the cerebellum [1]. Therefore, it would be expected that the cerebellum uses the same computational principle for simultaneous gain and timing control.

**Figure 1:**
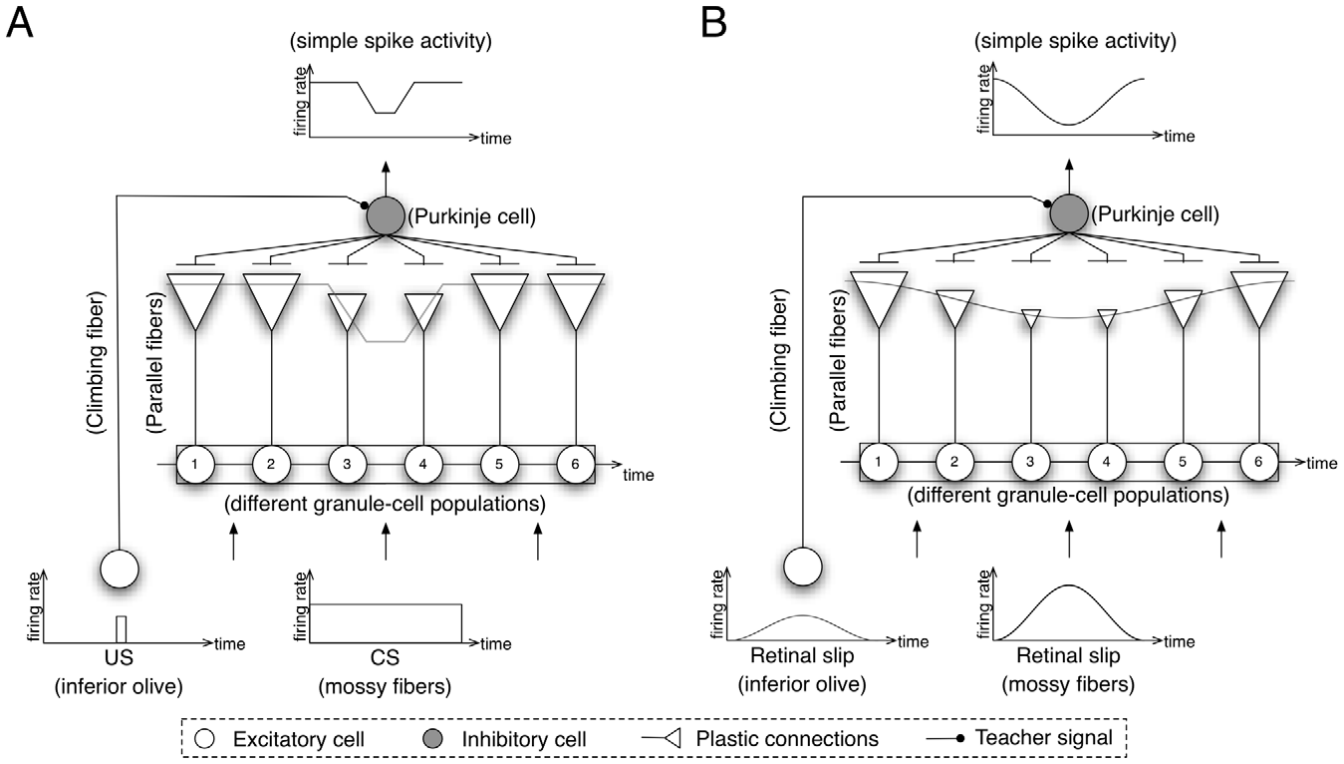
Hypothetical computational mechanism for (A) Pavlovian delay eyeblink conditioning and (B) gain adaptation of optokinetic response (OKR) eye movement. (A) In delay eyeblink conditioning, different populations of granule cells (numbered circles) become active one by one sequentially in response to a conditioned stimulus (CS) conveyed by mossy fibers (MFs). At the onset of an unconditioned stimulus (US) conveyed by a climbing fiber (CF), long-term depression (LTD) occurs only at the active parallel fiber (PF) synapses (synapses of granule-cell populations 3 and 4). Here, we depict the synapses as triangles at the end of PFs, and the size of them represents the efficacy of signal transmission from the PFs referred as “synaptic weights”. Consequently, the net input to a Purkinje cell (gray circle) decreases around the US onset, which causes a “pause” of the Purkinje cell's response. (B) In OKR adaptation, different populations of granule cells become active one by one sequentially in response to sinusoidally oscillating MF signals representing retinal slip information. LTD occurs by correlated firing of PFs and a CF, so that the spatial distribution of PF synaptic weights becomes sinusoidal. Therefore, the Purkinje cell's response modulates sinusoidally in time in a mirror-symmetric manner with the CF inputs.

In the previous study, we have proposed a cerebellar model for timing control [24]. Here, we extend the model to propose a single computational mechanism to unify gain and timing control. Figure 1 illustrates the hypothetical mechanism. In delay eyeblink conditioning (Fig. 1A), mossy and climbing fibers (MFs and CFs) convey respectively conditioned and unconditioned stimuli (CS and US). When a CS is presented, different populations of granule cells become active one by one sequentially and thereby representing the passage of time from the CS onset. At the US onset, long-term depression (LTD) occurs by correlated firing of the active parallel fibers (PFs) with the CF, by which the efficacy of signal transmission from the active PFs to the innervated Purkinje cells, referred as “synaptic weight” in this study, is decreased. Because the active PFs at the US onset is determined uniquely, and the synaptic weight only for the PFs is decreased, Purkinje cells gradually learn to pause around the US onset [28]. In OKR adaptation (Fig. 1B), MFs and CFs convey retinal slip information, which oscillates sinusoidally in time. From the start of a cycle of the sinusoidal oscillation, different populations of granule cells become active one by one sequentially. LTD shapes the spatial distribution of PF-Purkinje cell synapses sinusoidally, so that Purkinje cells' response gradually increases the depth of the sinusoidal modulation [29]. In this way, gain and timing control could be unified if Purkinje cells learn not the scalar information such as gain or timing but the complete waveform instructed by the CFs.

In order to justify our hypothesis, we adopted our large-scale spiking network model for delay eyeblink conditioning [25] to OKR adaptation, and conducted computer simulations. Our model was able to reproduce some of the electrophysiological findings in OKR adaptation experiments.

## Results

### Granule cell dynamics in response to sinusoidally oscillating MF signals

**Figure 2:**
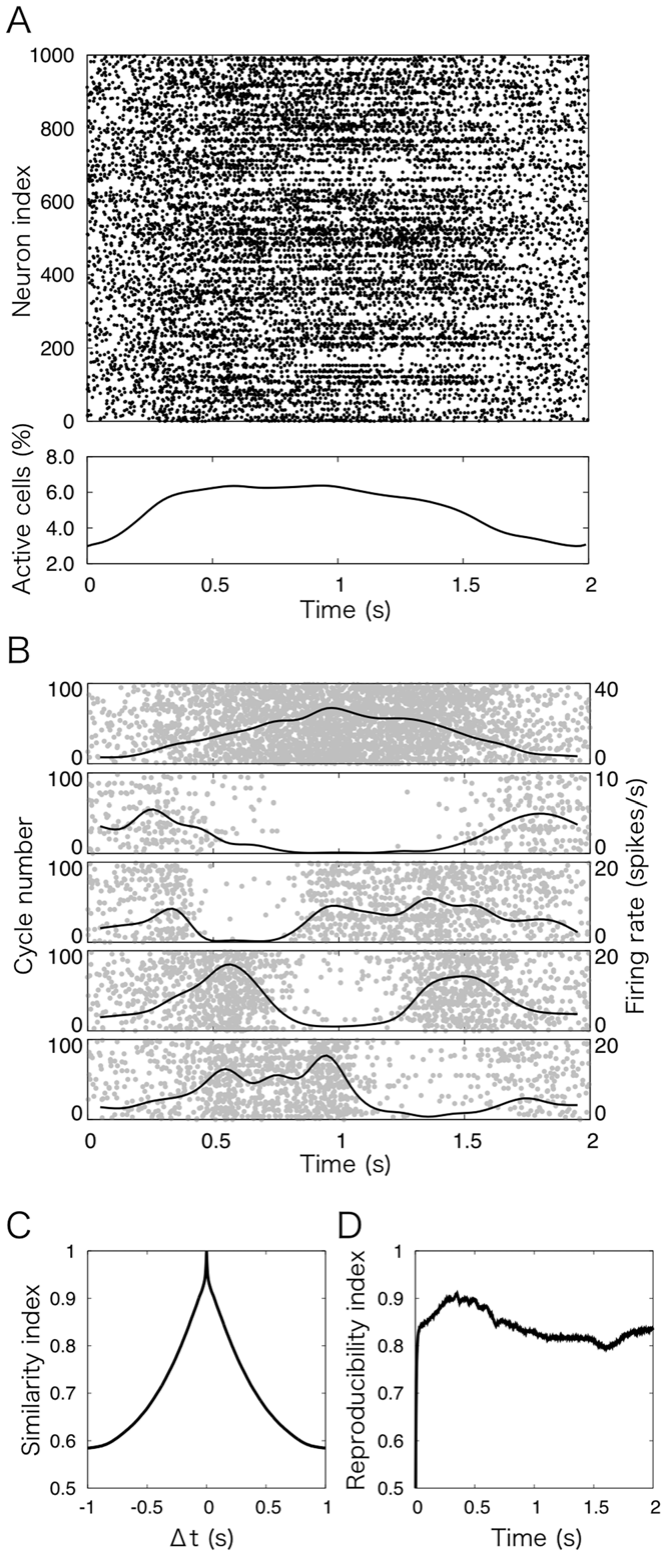
Dynamics of the granule cells in response to sinusoidally oscillating MF signals at 0.5 Hz. (A) Top panel, spike patterns of 1,000 out of 102,400 granule cells during a cycle of MF signal oscillation (2.0 s = 0.5 Hz). Black dots indicate spike discharges. At the beginning and end of a cycle where the firing rate of MFs is low, granule cells elicit spikes uniformly at random. At the middle of a cycle where the firing rate of MFs is high, spike patterns exhibit a variety of temporal profiles. Bottom panel, the ratio of active granule cells, which plots the number of active granule cells in each 10 ms bin divided by the total number of granule cells ( = 102,400). (B) Spike patterns of 5 representative granule cells across 100 cycles of MF signal oscillation. Black dots and grey lines in each panel show spike discharges in each cycle and the averaged spike density histogram, respectively. The granular layer modeled as a random recurrent network generates a variety of discharge patterns of granule cells, which can be used to produce a sequence of active granule-cell populations. (C) Similarity index *S*(Δ*t*) defined by Eq. (7) for the spike patterns shown in Figure 2A. The index monotonically decreases from 1 at Δ*t* = 0 to 0.58 as Δ*t* increases, suggesting that the active granule-cell population gradually changes into another, uncorrelated population over time. Therefore, a non-recurrent sequence of active granule-cell populations is generated. (D) Reproducibility index *R*(*t*) defined by Eq. (9) for 10 pairs of spike patterns for all granule cells across two successive cycles of MF signal oscillation. The reproducibility increases towards 0.9 at the beginning of a cycle, and then linearly decreases towards 0.8, suggesting that the spike patterns of granule cells are highly reproducible across cycles.

First, we examined how the granular layer generates a sequence of populations of granule cells in response to temporally oscillating, not temporally constant, MF inputs as in Figure 1B. To do so, we fed Poisson spikes that oscillate sinusoidally at 0.5 Hz to MFs as retinal slip signals exerted in OKR adaptation experiments [29], and examined the spike patterns of granule cells in response to the temporally oscillating MF inputs. The top panel of Figure 2A shows the spike patterns of 1,000 out of 102,400 granule cells in response to the sinusoidally oscillating MF signals at 0.5 Hz. At the beginning and end of a cycle of MF signal oscillation, where the firing rate of MFs is low, granule cells elicit spikes uniformly at random. As the firing rate of MFs increases, granule cells tend to sustain spike emission. The total activity of the granule cells is basically in proportional to the MF firing rate as shown in the bottom panel of Figure 2A, suggesting that the granule cells transmit the amplitude information of MFs to Purkinje cells. Yet, the waveform differs from that of MFs. The total activity rapidly increases during the first 0.3 s, and reaches the plateau where only 7% of the cells elicit spikes at each 10 ms bin. The plateau sustains for 0.7 s. Thereafter, the total activity slowly decreases towards the end of a cycle.

On the other hand, owing to the random recurrent connections between granule and Golgi cells, individual granule cells reveal a variety of temporal spike patterns as in Figure 2B, which depicts the spike patterns of 5 representative granule cells during 100 cycles of sinusoidally oscillating MF signals. The first two granule cells elicit spikes sinusoidally modulated with the same and the opposite phases, respectively. The next three granule cells exhibit more complex spike patterns: a pause for about 0.5 s starting from 0.4 s, 0.8 s and 1.0 s. Our previous computer simulations have demonstrated that temporally-fluctuating spike patterns of granule cells are generated in response to constant MF signals [25], [27]. The present study demonstrates that similar spike patterns are generated even by sinusoidally oscillating MF signals at 0.5 Hz.

These results imply that the population of active granule cells gradually changes in time. To confirm this property, we calculated the similarity index between active granule-cell populations separated Δ*t* by according to Eq. (7) and plotted in Figure 2C. The similarity decreases monotonically as Δ*t* increases, suggesting that the temporal change is nonrecurrent. This result guarantees the one-to-one correspondence between an active granule cell population and a time step from the beginning of a cycle of MF signal oscillation. In other words, a non-recurrent sequence of active granule-cell populations is generated as in Figure 1B.

Furthermore, Figure 2B implies that the generation of the temporally-fluctuating spike patterns are reproducible across cycles of MF signal oscillation. To confirm the reproducibility, we calculated the reproducibility index between two spike patterns for all granule cells for two consecutive cycles according to Eq. (9), and plotted it in Figure 2D. At 0.5 s within a cycle, the correlation is as high as 0.9, and then decreases almost monotonically towards 0.8, suggesting high reproducibility of the spike patterns across cycles. In general, the dynamics of a recurrent network depends on both the external inputs and the initial state of the network. The internal state needs to be reset at the beginning of each cycle so as to generate the same spike patterns across cycles. We will discuss how the sinusoidally oscillating MF signals reset the internal state of the granular layer in Discussion.

### Learned change of Purkinje cell and nuclear neuron responses

**Figure 3:**
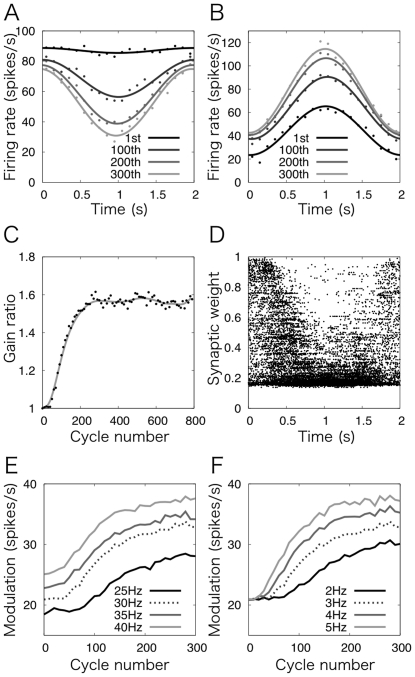
Simulation of OKR adaptation. (A and B) Learning-induced change of the firing of (A) a Purkinje cell and (B) a VN neuron at the 1st, 100th, 200th, and 300th cycles of MF signal oscillation (black to gray, respectively). The Purkinje cell increases the modulation from 3 to 24 spikes/s by decreasing the minimum firing frequency from 83 to 27 spikes/s. The maximal firing frequency changes modelately (from 89 to 75 spikes/s). The VN neuron increases the modulation from 21 to 33 spikes/s by increasing the maximal firing frequency from 63 to 121 spikes/s. The minimal firing frequency changes from 22 to 41 spikes/s. (C) Gain change with respect to the number of cycles. Gain ratio was defined by the modulation of a VN neuron at each 10 cycles divided by the modulation at the 1^st^ cycle. The gain ratio gradually increases and converges to 1.57 by 300 cycles. (D) The distribution of synaptic weights between active granule cells and Purkinje cells after 300 cycles of MF signal oscillation. The synaptic weights of active granule cells at the beginning and end of a cycle are uniformly distributed between 0.15 and 1, whereas these around the middle of a cycle are narrowly distributed between 0.15 and 0.5. Thus, the temporal change is reversely correlated with the waveform of the CF signal as in Figure 1B. (E and F) Modulation of a VN neuron by 300 cycles of MF signal oscillation with different peak firing rates of (E) MFs at 25, 30, 35 and 40 spikes/s while setting that of CFs at 3 spikes/s, and (F) CFs at 2, 3, 4 and 5 spikes/s while setting that of MFs at 30 spikes/s (black to gray). In both cases, the modulation increases as their peak firing rates increase.

Next, we examined how Purkinje cells change their activities and modify the activity of a neuron in the vestibular nucleus (VN) by learning. Figure 3A plots the firing of a Purkinje cell out of 16 Purkinje cells in the present model at the 1^st^, 100^th^, 200^th^, and 300^th^ cycles of MF signal oscillation. As the cycle number increases, the maximal firing rate changes moderately from 89 to 75 spikes/s, whereas the minimal firing rate decreases largely from 83 to 27 spikes/s. Hence, the modulation of the cell's firing, which is defined as the maximal minus minimal firing rates divided by 2, gradually increases across cycles from 3 to 24 spikes/s. This dynamics, in which the increase of the modulation of Purkinje cells' firing is mainly caused by the decrease of the minimal firing rate, is consistent with the change of Purkinje cells' simple spike firing in OKR experiments [29].

Figure 3B plots the firing of a neuron in VN, which is innervated by all 16 Purkinje cells in the model, at the same cycles. Because the inhibition exerted by the Purkinje cells modulates out-of-phase, the modulation of the VN neuron's firing becomes in-phase with MF signal oscillation, and increases gradually across cycles from 21 to 33 spikes/s. We conducted the simulation for 800 cycles and plotted the gain ratio (modulation of the VN neuron's firing at each cycle divided by that at the 1^st^ cycle) in Figure 3C. Owing to the inhibition of the inferior olive (IO) exerted by VN, we found that learning is saturated by the first 300 cycles of MF signal oscillation, and the gain ratio converges to 1.57. Therefore, we limited further analysis to the first 300 cycles.

Figure 3D plots the distribution of synaptic weights of active granule cells at each time step after 300 cycles of MF signal oscillation. Synaptic weights of active granule cells at the beginning and end of a cycle are uniformly distributed within 0.15 to 1.0. Around the middle of a cycle, where the amplitude of MF and CF signals is the largest, the distribution is localized between 0.15 to 0.5, and only a very small fraction of active granule cells have large synaptic weights. Thus, the temporal distribution of synaptic weights sinusoidally modulates out-of-phase with MF and CF signal oscillation, as we hypothesized in Figure 1B. In other words, the distribution of the synaptic weights represents the complete waveform of the CF signals in upside down. On the other hand, we observed that blocking feedforward inhibitory inputs to Purkinje cells exerted by basket cells resulted in in-phase modulation of Purkinje cells' firing, which is consistent with experiments [30]. This result suggests that the out-of-phase modulation of Purkinje cells' firing is caused by the spatial distribution of PF-Purkinje cell synaptic weights as well as the feedforward inhibition by basket cells.

We also carried out the same simulations for 300 cycles by varying the peak firing rate of MFs at 25, 30 (default), 35 and 40 spikes/s while setting that of CFs at 3 (default) spikes/s, and by varying the peak firing rate of CFs at 2, 3 (default), 4 and 5 spikes while setting that of MFs at 30 (default) spikes/s. Figure 3E and 3F plot the modulation of a VN neuron with respect to the firing rate of MFs (Fig. 3E) and CFs (Fig. 3F). The modulation of the VN neuron increases as their firing rates increase, which corresponds to that both the MF and CF inputs represent retinal slip information in OKR in the present model. Specifically, by increasing the peak firing rate of MFs, the excitatory inputs to a VN neuron become stronger, whereas the inhibitory inputs do not change largely because the Purkinje cells' activity is regulated by both the PFs and inhibitory interneuons. Therefore in Figure 3E, the modulation of a VN neuron at zero cycle is proportional to the peak firing rates of MFs. On the other hand, in Figure 3F, by increasing the peak firing rate of CFs, the inhibitory inputs through Purkinje cells become weaker owing to LTD, whereas the excitatory inputs through MFs remains the same. Thereby, the modulation of a VN neuron increases proportionally with the peak firing rate of CFs, whereas the modulation at zero cycle is the same. Moreover, larger firing rate of CFs induces more LTD, resulting in more remarkable increases of the modulation of a VN neuron.

### Frequency-dependent increase of nuclear neuron firing

Previous experiments for OKR adaptation in rodents and VOR adaptation in monkeys and rodents consistently demonstrated frequency-dependent gain increase [31]–[33]. In the experiments of OKR gain adaptation, continuous screen oscillation at a fixed frequency is used for training. The gain increase is found to be the largest at the oscillation frequency which was used for training, whereas a small gain increase is still observed for other frequencies. The underlying neural mechanisms of such frequency-dependent gain increase, however, are not well known.

**Figure 4:**
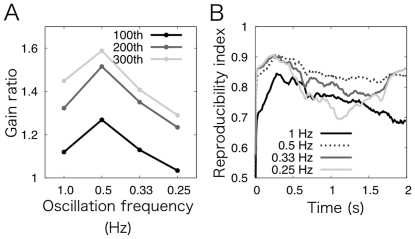
Frequency-dependence of gain change in OKR adaptation. (A) The gain ratio defined as in Figure 3C at the test frequencies of 1 Hz, 0.5 Hz ( = training frequency), 0.33 Hz and 0.25 Hz at the 100^th^, 200^th^ and 300^th^ cycle (black to gray, respectively). The gain ratio increases as the cycle number increases. The ratio is the largest when the test frequency is equal to the training frequency, suggesting frequency-dependent change of OKR gain. (B) Reproducibility indices between 10 pairs of spike patterns for all granule cells in response to MF signal oscillation with the frequency of 0.5 Hz and those with the frequencies of 1 Hz, 0.5 Hz, 0.33 Hz and 0.25 Hz (black, dotted, gray, and pale gray lines, respectively). Spike patterns are normalized in length to 2 s ( = 0.5 Hz) by renumbering the time step. The reproducibility indices for non-training oscillation frequency are lower than that for training oscillation frequency, suggesting worse reproducibility of spike patterns in response to MF signal oscillation with non-training oscillation frequencies.

To investigate whether our model shows similar frequency-dependent gain increase, we conducted a computer simulation for 300 cycles of MF signal oscillation with the training frequency of 0.5 Hz while saving the learned information, namely the synaptic weights at PF-Purkinje cell synapses to a storage. Then, we conducted additional simulations with different test frequencies (0.25 Hz, 0.33 Hz, 0.5 Hz and 1 Hz) using the stored synaptic weights at the 100, 200 and 300^th^ cycle during the training as the initial synaptic weight. We measured the modulation of a VN neuron's firing and calculated the gain ratio in Figure 4A. The gain ratio increases as the cycle number increases irrespective of the test frequencies, but the ratio is the largest at the test frequency of 0.5 Hz. Therefore, our network model reproduces the frequency-dependent generalization of VN neuron firing.

**Figure 5:**
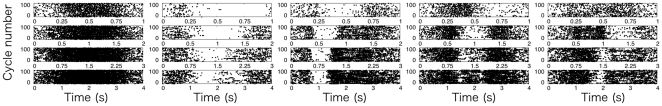
Spike patterns of 5 representative granule cells shown in **Figure 2B** **for 100 cycles of MF signal oscillation with frequencies of 1 Hz, 0.5 Hz (default), 0.33 Hz and 0.25 Hz (top to bottom, respectively).** Abscissa represents the duration of a cycle, so that the duration varies at 1, 2, 3 and 4 s, respectively. Throughout the test frequencies, similar spike patterns are generated while temporally expanded or compressed.

What computational mechanism underlies the frequency dependence? We hypothesized that the granular layer plays an essential role, and further examined the spike patterns of granule cells in response to MF signals oscillating at these four test frequencies. We normalized the spike patterns by rescaling the duration of a cycle to 2.0 s ( = 0.5 Hz). Then, we calculated the reproducibility index between the original spike patterns for all granule cells at 0.5 Hz of MF signal oscillation and a normalized spike patterns generated at different test frequencies. Figure 4B shows the result. The reproducibility is still high irrespective of the test frequencies. The best reproducibility, however, is obtained when the test frequency is equal to the training frequency (i.e., 0.5 Hz). This observation implies that the high reproducibility at all test frequencies causes the general increase of the gain ratio, whereas the frequency-dependent reproducibility results in the frequency-dependent increase of the gain ratio. We also plotted the spike patterns of the representative 5 granule cells shown in Figure 2B in response to MF signal oscillation with these different frequencies across 100 cycles in Figure 5. These spike patterns show similar temporally-fluctuating profiles of the firing, except for that the spike patterns are temporally expanded or compressed depending on the frequency. These observations imply that the granular layer generated the same spike pattern in response to MF signals while temporally compressing or expanding the spike pattern for different frequencies. In sum, we suggest that the frequency dependence of gain increase emerges from the network dynamics of the granular layer rather than the training frequency selective channels [31].

## Discussion

In the present study, we proposed a computational mechanism to unify the cerebellar gain and timing control. Specifically, we demonstrated that a class of cerebellar timing control models, in which a recurrent network as a model of the granular layer generates a non-recurrent sequence of active granule-cell populations that can represent a passage of time, can be adopted also for gain control. The first theoretical model of this class was proposed by Buonomano and Mauk (1994) [17], and later more elaborated model was implemented by Medina et al. (2000) [20] so as to compare the simulation results quantitatively with their experimental data for Pavlovian delay eyelid conditioning. We formulated the recurrent network, and theoretically and numerically analyzed its dynamics [24]. Later, we implemented our theoretical model into a large-scale spiking network, which can act as a general supervised learning machine known as a liquid state machine [25], [27], [34]. In the present study, we adopted the spiking network model to OKR adaptation and succesfully reproduced the learning-induced change of simple spike firing of Purkinje cells, learning-induced gain increase, and frequency-dependent gain increase.

### Properties of the sequence generation of active granule-cell populations

A key assumption of the present model is that the granular layer, modeled as a recurrent inhibitory network composed of granule and Golgi cells, generates a nonrecurrent sequence of active granule-cell populations. In our previous modeling study [25], we reported that such a sequence could be generated in response to sustained MF inputs in which the firing rate is constant at 30 spikes/s. In the present study, we suggested that a similar sequence can be generated even for the sinusoidally oscillating MF inputs at 0.5 Hz (Fig. 2). The granular layer has to generate the same sequence of active granule-cell populations across different trials or cycles to reliably transmit MF information. Because the dynamics of a recurrent network depends on the external inputs as well as the initial state of the network, the granular layer has to reset its internal state at the beginning of each trial or cycle for this purpose. In our previous simulations for delay eyeblink conditioning [25], we provided a brief strong input (200 spikes/s for 5 ms) at the CS onset, which should activate most granule cells instanteneously, thereby resetting the internal state of the granular layer. In the present study, we did not provide such strong inputs. Rather, we demonstrated that slowly increasing MF signals may be enough to reset the internal state, as shown by the reproducibility index in Figure 2D. The reset mechanism could be explained as follows. Figure 2B shows that the rising phase of granule cells' activity sustains for 0.3 s at the beginning of each cycle. In this phase, increase of excitatory MF inputs to granule cells advances temporally that of feedback inhibition exerted by Golgi cells. The dominance of excitation produces a situation in which most of the granule cells are activated uniformly. This uniformly-activated state may serve as a default internal state that occurs at the beginning of each cycle. Therefore, slowly increasing excitatory inputs are sufficient to reset the network dynamics. In VOR, Barmack and Yakhnista (2008) have reported the presence of phasic components of MF signals at the onset of the head rotation [35]. Such phasic components may exist in OKR as well.

Recently, Jirenhed and Hesslow (2011) have reported that in the preparations of fully conditioned decerebrated ferrets, a brief CS consisting of only one or two impluses in MFs is sufficient to elicit a pause in 4 out of 7 Purkinje cells recorded [36]. Because such a brief CS seems to be unable to evoke a sequential activation of granule-cell populations, they suggest that a traditional view of the cerebellar timing mechanisms including ours, in which different parallel fibers are activated with different temporal patterns [14]–[17], [19]–[22], [24], [25], [27], may be unlikely. However, in brainstem-cerebellum preparations of turtles, a brief stimulation of MFs evokes long-lasting excitatory postsynaptic potentials (EPSPs) that sustain up to 800 ms in Purkinje cells [37]. Importantly, these EPSPs are blocked by the bath application of DL-2-amino-5-phosphonvalerate, indicating the contribution of *N*-methyl-*D*-aspartate receptors (NMDARs) at MF-granule cell synapses [37]. We have suggested that NMDARs at MF-granule cell synapses are indispensable for the generation of a sequence of active granule-cell populations [25], [27]. These results imply that even a brief stimulation of MFs could evoke sustained activity of granule cells, thereby generating a sequential activation of granule-cell populations. We will extend the present model to incorporte these findings in future.

Electrophysiological studies have shown that granule cells excite Golgi cells, which in turn inhibit granule cells, thereby constituting a recurrent network. Yet, whether such a network actually works as a random recurrent network that generates a non-recurrent sequence of granule-cell populations remains unknown. Detailed analysis of neural activity of the granular layer and reproduction in a computer simulation would be necessary to clarify this issue.

### Mechanism underlying the stimulus-frequency dependency of adaptation

We also observed that in response to MF inputs with different oscillation frequencies, similar spike patterns of granule cells are generated while being expanded or compressed temporally as shown in Figure 5. Because the granular layer is modeled as a random recurrent network that generates spike patterns in a highly nonlinear manner, the network could generate very different spike patterns in response to oscillatory MF inputs with different frequencies. Therefore, generation of the temporally expanded/compressed spike patterns is not trivial. We consider that this property may result in the frequency-dependent gain increase observed in experiments [31]–[33]. Furthermore, this result implies that the internal representation of a passage of time by the sequential activation of granule-cell populations can be sped up or slowed down adaptively depending on the frequency or the amplitude of MF inputs. Experiments of delay eyeblink conditioning have shown that the learned CR can be elicited earlier or later than the trained timing, when the amplitude of a CS is respectively increased or decreased, suggesting that the learned timing may be expressed adaptively depending on the amplitude of MF inputs [38]. We hypothesize that the adaptive change of the learned timing is caused by the adaptive speed up/slow down of the internal representation of a passage of time.

### Comparison of model granule cells' activity with experimental data

The present model assumes that individual granule cells elicit spikes in random and intermitted manner so as to generate a nonrecurrent sequence of active granule-cell populations (Fig. 2B). Unfortunately, recent experiments of in-vivo granule cell recording report that granule cells elicit spikes regularly and faithfully in response to peripheral or direct MF stimulation. Here, we would like to interpret this discrepancy. Some studies [35], [39]–[41] used ketamine for anesthesia, which is a blocker of NMDARs ([42] and references therein). As already mentioned, long decay time constants of NMDAR-mediated EPSPs are indispensable for the generation of a sequence of active granule-cell populations. Two studies [39], [40] used very brief sensory stimulation that sustains for 50 ms. In delay eyeblink conditioning, the inter-stimulus interval (ISI) should be >100 ms to generate robust CRs [3], suggesting that the sensory stimulation used in their experiments were too brief to observe the spatiotemporal dynamics of granule-cell activity proposed in the present model. The other studies using awake animals [43], [44] displayed granule-cell activity in peristimulus time histograms using a relative large bin size, so that the fine temporal structure within the granule-cell activity might be hidden. Single-unit recordings of granule cells from awake animals in finer temporal resolution may be helpful to examine the activity of granule cells suggested in the present study.

### Unification of gain control and timing control models

The present model for OKR adaptation has inhibitory projection from VN to IO, although such inhibitory projection has not been demonstrated experimentally. This inconsistency is due to that the present model is adopted from our previous model for delay eyeblink conditioning [25], in which inhibitory projection from the cerebellar nuclei to IO has been reported [45]. We would like to consider why the inhibitory projection exists from cerebellar nuclei to IO, which does not from vestibular nuclei to IO, from a functional point of view. In the case of delay eyeblink conditioning, the US is fed forcibly by an experimenter. In other words, “error” signal always comes into IO from the peripheral no matter how long the training proceeds. This may cause overtraining, but the inhibitory projection from the nuclei to IO can work to suppress it (Fig. 3C). On the other hand, in the case of VOR/OKR adaptation, retinal slip would gradually decreases during the training, suggesting that error signal naturally diminishes without any external mechanisms. Therefore, the inhibitory projection would be no longer necessary and become very small.

### Comparison with other models

Adaptive filter models of the cerebellum are another candidate for simultaneous gain and timing control (e.g., [46]). In the phase converter model [5] for VOR and OKR adaptation, the activity of individual granule cells are oscillated with different phases in response to sinusoidally modulating MF signals. The phase converter model assumes that the phase differences of granule cells' activity are generated by the sum of the MF signal and the inhibition exerted by the Golgi cell acting as an integrator. The oscillator model for the delay eyeblink conditioning [14] extends this assumption, therein the activity of individual granule cells are oscillated with different frequencies and phases in response to constant MF signals. The computation of these two models is mathematically equivalent to the Fourier series expansion, which represents a given waveform by a combination of sinusoids. Another variant of adaptive filter models [11], in which individual granule cells are activated one-by-one sequentially as in the tapped delayline model [47], has been proposed and adopted to VOR adaptation. Throughout these three adaptive filter models, individual granule cells are regarded as as a set of temporal “filters”. Two differences are noted between adaptive filter models and our model. First, adaptive filter models assume that individual granule cells encode the information conveyed by MFs. On the other hand, our model assumes that populations of granule cells, not individual cells, encode the information. Second, adaptive filter models do not explain how these filters are implemented biologically within the recurrent network of the granular layer. A calculation shows that granule cells in the phase converter model could exhibit only temporally-constant activities in response to constant MF signals, and the latter two models [11], [14] assume that the filters are given a priori. On the other hand, our model employs an identical granular layer network to generate temporally-fluctuating spike patterns of granule cells for both sinusoidally modulating and constant MF signals. Summarizing, the difference between the adaptive filter models and our model is the modeling of the granular layer.

The Marr-Albus-Ito's model of a simple perceptron provides a general theory of cerebellar computation [1], [48], [49]. In their model, external input signals are represented sparsely and combinatorially by MFs (codon representation) and/or granule cells (expansion recoding), so that a variety of input patterns can be encoded efficiently. In our model, spatiotemporal information conveyed by MFs are recoded into sparse spatiotemporal spike patterns in much higher dimensions by a large number of granule cells. Therefore, our model can be regarded as a natural extention of their model into temporal domain. Theoretically, the present model can aquire the relationship between arbitrary spatiotemporal signals fed by MFs and CFs as “context” and “desired” signals, respectively. This ability to learn and replay arbitrary desired signals in response to given contextual information may be essential for acquiring “internal models” that simulate the dynamics of a diversity of physical and mental objects [50].

In sum, we proposed a computational mechanism that unifies gain and timing control mediated by the cerebellum. We carried out large scale computer simulations of a spiking network model of the cerebellum to justify our hypothesis. This study may shed light on a universal computational principle employed by the cerebellum.

## Materials and Methods

### Implementation of the present cerebellar model

#### Network structure

**Figure 6:**
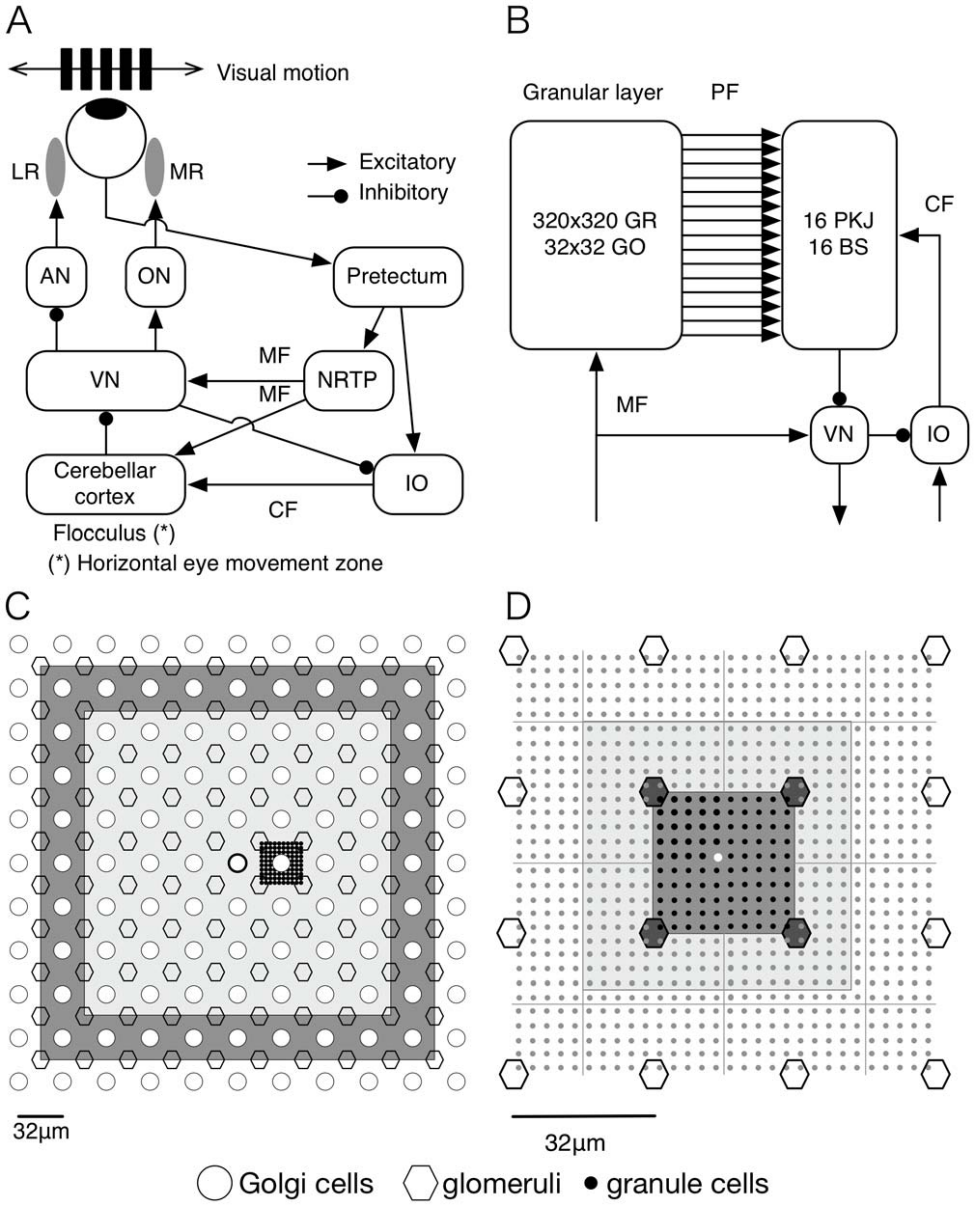
Schematics of the neural circuitry for horizontal OKR adaptation in rabbits (A), the structure of the model cerebellum (B, C, D). (A) The VN receives the visual motion information through MFs via the pretectum and NRTP, and drives the extraocular muscles (AN and ON) to evoke OKR. The cerebellar cortex (specifially, horizontal eye movement zone of the flocculus) receives the visual motion information through MFs and CFs, and inhibits the VN. The VN also inhibits the IO which sends CFs to the cerebellar cortex. (B) MF inputs are fed to model granule cells in the model granular layer of the cerebellar cortex and the model VN neuron. Model granule cells excite nearby model Golgi cells, which in turn inhibit nearby model granule cells. The model granule cell activity is fed to model Purkinje cells and basket cells via PFs. Model basket cells inhibit nearby model Purkinje cells, and all Purkinje cells inhibit the model VN. Model Purkinje cells also receive CFs. The model VN issues the final output, and inhibits the model IO. (C) Spatial arrangement of model Golgi cells (circles), glomeruli (hexagons) and granule cells (dots). Shaded rectangles in pale and dark grey represent, respectively, the dendritic and the axonal arborization of the model Golgi cell at the center. (D) Spatial arrangement of model glomeruli (hexagons) and granule cells (dots) in detail. The rectangle in pale grey represents the dendritic arborization of model granule cell at the center (white dot). For example, 100 model granule cells in the grey box (black dots) contact the four simulated glomeruli surrounding these model granule cells (filled hexagons). Panels C and D are taken from [25]. Abbreviations: AN, abducens nucleus; BS, basket cell; CF, climbing fiber; GO, Golgi cell; GR, granule cell; IO, inferior olive; LR, lateral rectus muscle; MF, mossy fiber; MR, medial rectus muscle; NRTP, nucleus reticularis tegmentis ponti; ON, oculomotor nucleus; PF, parallel fiber; PKJ, Purkinje cell; VN, vestibular nucleus.

Figure 6A illustrates a schematic of the neural circuit involved in horizontal OKR adaptation [51]. Visual motion information is transmitted from the retina to both the horizontal eye movement zone of the flocculus and VN via the pretectum and nucleus reticularis tegmenti pontis (NRTP) through MFs. Visual motion information is also transmitted to the flocculus through CFs via the IO, which receives inputs from the pretectum. The flocculus inhibits the VN, which in turn drives the extraocular muscle motor neurons. In the present study, we modeled the cerebellar cortex (flocculus), VN, IO, and MFs to focus on the dynamics of the flocculus and VN.

The model cerebellar cortex (Fig. 6B) is composed of the model granular layer with 320
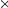
320 model granule cells and 32
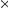
32 model Golgi cells aligned on two-dimensional grids, 16 model Purkinje cells and the same number of model basket cells aligned parasagittally [25]. Each model granule cell has 4 dendrites, receives excitatory inputs from MFs and receives inhibitory inputs from model Golgi cells via simulated glomeruli (Fig. 6C). A model granule cell contacts the 4 nearest simulated glomeruli resulting in a nested structure of the granular layer due to the square arrangement of model granule cells: 10
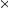
10 model granule cells contact the same simulated glomeruli, resulting in “granule-cell clusters” (Fig. 6D). Thus, 100 model granule cells in a cluster share the same excitatory and inhibitory inputs. A simulated glomerulus receives inhibitory inputs from 9
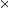
9 nearby model Golgi cells with probability 0.025, so that, on average, 2 model Golgi cell axons innervate a simulated glomerulus. Hence, a model granule cell receives, on average, 8 inhibitory inputs through 4 dendrites. A glomerulus also receives an MF input, so that model granule cells contacting the same glomerulus should receive a common excitatory signal. However, to simulate the stochastic variability of synaptic transmission from a glomerulus to granule cells, we modeled individual granule cells contacting the same glomerulus to receive different Poisson spikes with the same firing frequency (see “Stimulus” section for details). On the other hand, a model Golgi cell receives inputs from 700
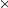
700 model granule cells, namely 7
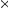
7 granule-cell clusters with probability 0.05.

A model Purkinje cell and a model basket cell receive PF inputs from 9
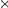
32 granule-cell clusters. A model Purkinje cell also receives inhibitory inputs from 3 nearby model basket cells and CF inputs from a model IO. A model VN neuron receives excitatory inputs from 100 MFs as well as inhibitory inputs from all model Purkinje cells, and issues the final output of the model cerebellum while inhibiting the model IO neuron. Although inhibitory projection from VN to IO has not been demonstrated experimentally, our model incorporates this projection, because this model is based on our previous model for the delay eyeblink conditioning, in which inhitory projection from the cerebellar nuclei to IO has been reported [45]. The model IO neuron provides CF inputs to model Purkinje cells, which trigger simulated plasticity at PF-Purkinje cell synapses. The present model does not consider CF collateral inputs to the VN, because they are not present in all animal species [52].

#### Neuron model

Neurons are modeled as conductance-based leaky integrate-and-fire units [53]:
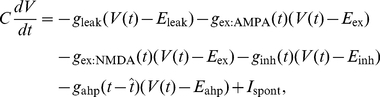
(1)where *V*(*t*) and *C* are the membrane potential at time *t* and the capacitance, respectively. The membrane potential is determined by six types of currents specified by Eq. (1); namely, leak, AMPAR (α-amino-3-hydroxy-5-methyl-4-isoxazolepropionic acid receptor)-mediated currents, NMDAR-mediated currents, GABA_A_R (γ-aminobutyric acid type A receptor)-mediated currents, the current for emulation of the after-hyperpolarization, and the external current for simulating spontaneous discharge. For each type *c* ∈ {leak, ex:AMPA, ex:NMDA, inh, ahp}, the current at a given time is calculated with conductance *gc* and reversal potential *Ec* . The conductance is calculated by the convolution of the exponential function α(*t*) and the spike event δ*j*(*t*) of presynaptic neuron *j* at time *t* as follows:
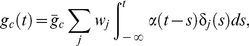
(2)where 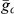 represents the maximum conductance and *w_j_* the efficacy of signal transmission referred as synaptic weight from the presynaptic neuron *j*. The exponential functions are defined for each current and each neuron type with different time constants. When the membrane potential of a neuron exceeds the threshold *θ*, the neuron elicits a spike, which is followed by the after-hyperpolarization that determines a refractory period. The conductance for the after-hyperpolarization is given by
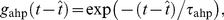
(3)where 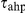 represents the time constant of the after-hyperpolarization, and 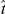 is the last firing time of the neuron [53]. The external current for simulating spontaneous discharge (*I*_spont_) is fed to only model Purkinje cells and a VN neuron, because these neurons are known to maintain relatively high spontaneous discharge rates [54]–[57]. Parameter values were adopted from known physiological data (Tables 1 and 2) as in our previous model [25], except for the cell parameters for model basket cells, the exponential function representing inhibitory postsynaptic potentials (IPSPs) at model Purkinje cells, and the external currents.

**Table 1:** Summary of cell parameters.

Cell parameters		Cell type					
		GR	GO	PKJ	BS	VN	IO
	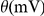	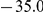	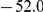	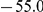	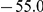	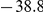	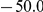
	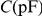	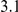	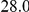	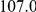	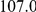	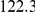	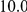
	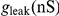	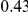	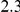	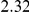	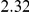	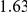	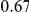
	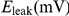	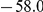	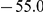	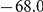	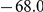	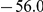	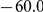
	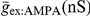	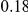	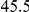	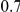	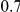	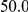	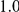
	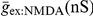	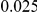	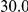	−	−	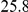	−
	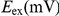	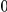	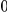	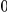	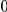	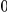	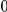
	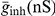	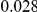	−	1.0	−	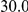	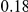
	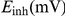	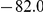	−	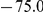	−	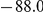	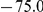
	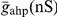	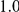	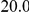	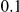	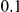	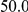	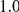
	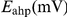	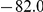	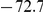	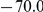	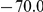	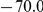	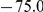
	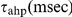	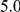	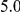	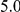	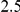	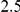	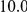
	*I*_spont_ (nA)	−	−	0.25	−	0.7	−

Abbreviations: GR, granule cell; GO, Golgi cell; PKJ, Purkinje cell; BS, basket cell; VN, vestibular nuclear neuron; IO, inferior olivary neuron; −, nonexistent.

**Table 2:** Summary of exponential functions.

GR	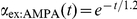
	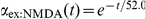
	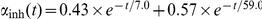
GO	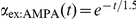
	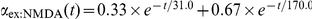
PKJ	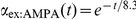
	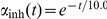
BS	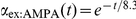
VN	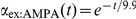
	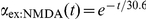
	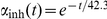
IO	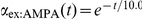
	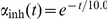

All units are in millisecond. Abbreviations: GR, granule cell; GO, Golgi cell; PKJ, Purkinje cell; BS, basket cell; VN, vestibular nuclear neuron; IO, inferior olivary neuron.

#### Stimulus

We used a fictious sinusoidal oscillation of check-patterned screen at 0.5 Hz (one cycle) to simulate visual stimulus, by referring to the experiments of OKR adaptation (e.g., [50]). We assumed that model NRTP neurons elicit spikes in response to the sinusoidal fictious screen oscillation with spontaneous and maximum firing rates of 15 and 30 spikes/s, by referring to data in rabbits [58]. We modeled MF signals as Poisson spike trains whose probability distribution modulates sinusoidally at 0.5 Hz with the mean and maximum amplitudes of 15 and 30 spikes/s, respectively. We neglected the gradual decrease of MF signals induced by learning, because sufficient retinal slip still remains even after the end of OKR training under the relatively fast (0.5 Hz) screen oscillation. This means that all the simulations conducted in the present study are open loop. We also modeled CF signals as sinusoidally modulating spike trains with the mean and maximum amplitudes of 1.5 and 3 spikes/s, respectively. To obtain such spike patterns, we fed Poisson spike trains that modulates sinusoidally with the equivalent mean and maximum amplitude to the IO. We neglected the gradual decrease of CF signals induced by learning as well. These values for MFs and CFs are consistent with data in rabbits [29].

Because the input to IO means retinal slip information in OKR, first we fed to the IO the same Poisson spikes as MFs with the peak firing rate of 30 spikes/s. We, however, realized that it was impossible to obtain the peak firing rate of CFs as low as 3 spikes/s by the input. Eventually, we decided to decrease the peak firing rate of the input to 3 spikes/s so as to obtain the 3-spikes/s peak firing rate of CFs. Several reasons would exist for the low firing rate of CFs. Electrophysiological studies have shown that neurons in IO are electrically coupled and oscillates at about 10 Hz under the threshold. The subthreshold oscillation could work as a temporal filter by which input signal to IO are passed to Purkinje cells only when the signal comes into IO at the same timing that the oscillation reaches the peak [59]. This mechanism could reduce the firing rate of CF dramatically.

#### Synaptic weights

Synaptic weights incorporated in Eq. (2) are set identically as in our previous model [25], except for those related to Purkinje cells and basket cells that were missing in the previous model. Criteria for setting parameters are described in [25], but briefly, the parameters are set so as to achieve a maximal firing rate near 100 spikes/s for each model neuron. We found that the present model is able to reproduce similar results within a wide range of parameters, suggesting that careful parameter settings are not necessary. The parameter values we used in the present study are shown in Table 3.

**Table 3:** Summary of synaptic weights.

	Postsynaptic neuron							
Presynaptic neuron		MF	GR	GO	PKJ	BS	VN	IO
	MF	−	4.0	−	−	−	0.002	−
	GR	−	−	0.00004	0.003	0.003	−	−
	GO	−	10.0	−	−	−	−	−
	PKJ	−	−	−	−	−	0.008	−
	BS	−	−	−	5.3	−	−	−
	VN	−	−	−	−	−	−	5.0
	IO	−	−	−	1.0	−	−	−

Abbreviations: GR, granule cell; GO, Golgi cell; PKJ, Purkinje cell; BS, basket cell; VN, vestibular nuclear neuron; IO, inferior olivary neuron; −, nonexistent.

#### Plasticity

The plasticity rule for PF-Purkinje cell synapses are the same as in our previous study [25]. The synaptic weight between model granule cell *j* to model Purkinje cell *i* at time *t*, denoted by *w*_PKJ*i* ←PF*j*_ (*t*), is updated as follows:
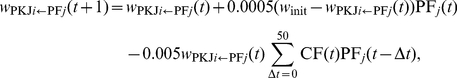
(4)where PF*j* (*t*) and CF(*t*) take 1 if PF*j* or CF elicited a spike at time *t* , and 0 otherwise. The 2nd term on the right-hand side simulates long-term potentiation (LTP) [60]–[62] by PF stimulation only, whereas the 3rd term LTD [63] by conjunctive activation of a CF and a PF that are active 0–50 ms earlier than the CF activation. The constant denoted by *w_init_* represents the initial synaptic weight and was set at 1.0. We have tested a larger time window up to 250 ms according to slice experiments [64], and still obtained similar results by setting the coefficient for LTP at a larger value.

### Analysis of the spike patterns of granule cells

In order to study how the activity pattern of model granule-cell clusters evolved over time, we defined several indices as in our previous study [25]. First, we defined the population average activity of model granule-cell cluster *i* at time *t* as
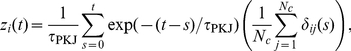
(5)where *N_c_* is the number of model granule cells in a cluster ( = 100), δ*_i j_* (*t*) takes a value of 1 if granule cell *j* in the cluster *i* elicited a spike at time *t* while a value of 0 otherwise, and *τ*_PKJ_ represents the time constant of AMPAR-mediated EPSPs at PF-Purkinje cell synapses ( = 8.3 ms). In other words, *z_i_* (*t*) represents the AMPAR-mediated EPSPs at a model Purkinje cell evoked by the *i* th model granule-cell cluster at *t*. We then defined the autocorrelation of the activity pattern at times *t* and *t* +Δ*t* as follows:
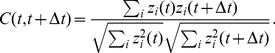
(6)The numerator represents the inner product of population vectors of model granule-cell clusters at times *t* and *t* + Δ*t*, and the denominator normalizes the vector lengths. Because *z_i_* (*t*) takes only positive values, the correlation takes a value between 0 and 1. The correlation would be 1 if the population vectors at time *t* and *t* + Δ*t* are identical, and 0 if the vectors are orthogonal, indicating that the active populations have no overlap.

We then defined the similarity index *S*(Δ*t*) as follows:
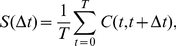
(7)where *T* represents the inverse of the oscillation frequency of MF inputs ( = 2 s). This is the average of Eq. (6) with respect to *t*. This index represents how two population vectors separated by Δ*t* are correlated, on average. For example, given a sequence of populations of granule cells in which the same population appears repeatedly in time, *S*(Δ*t*) oscillates with respect to Δ*t*, and vice versa. Therefore, if the similarity index decreases monotonically as Δ*t* increases, then the sequence of granule-cell populations are not periodic, indicating that an active population evolves with time into uncorrelated populations.

We also studied the reproducibility of the activity pattern of model granule-cell clusters across different cycles of MF signal oscillation. First, we calculated the correlation of the activity patterns at the *k* th and the successive cycles {*z_i_* ^(*k*)^(*t*)} and {*z_i_* ^(*k*+1)^(*t*)} at time *t*:
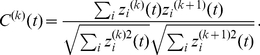
(8)We then defined the reproducibility index *R*(*t*), which indicates how two activity patterns are differentiated in time, as the average of the correlation with respect to successive *K* cycles follows:
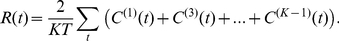
(9)We typically used 10 pairs of successive cycles (*K* = 20) to calculate *R*(*t*).

### Simulation of OKR adaptation

For simulating OKR adaptation, we repeated 300 cycles of the simulated screen oscillation to train the model cerebellar network. That is, MF inputs and IO inputs are fed for 300 cycles, and at each cycle, PF-Purkinje cell synaptic weights are updated according to Eq. (4). After each cycle, we carried out 10 additional cycles of the simulated screen oscillation separately and calculated the firing rate of model Purkinje cells and a VN neuron. During these 10 cycles, PF-Purkinje cell synaptic weights are not updated, so as to avoid unnecessary learning.

### Software

The program was written in C with GNU Scientific Library (GSL). Differential equations were solved numerically using the 4th order Runge-Kutta method contained in GSL with a fixed step time of 1 ms. Data analysis was performed by custom software written in Ruby language. We will release the source code upon acceptance at the public database Cerebellar Platform [65].
